# Autonomous cameras reveal larval reef fish responses to acoustic enrichment and lunar phase

**DOI:** 10.1038/s41598-026-57857-9

**Published:** 2026-06-26

**Authors:** Océane Boulais, Aaron Thode, Corinne Pickering, Natalie Levy, Daniel Schar, Jessica Reichert, Tyler Maldonado, Josh Madin, Joshua Levy, R3D Consortium, Ben Jones, Sean Mahaffey, Aricia Argyris, Mark Aruda, Ian Robertson, Zhenhua Huang, Ayrton Medina-Rodriguez, Mert Gokdepe, Brady Halvorson, Jon Chase, Charlotte White, Cami Dillon, Kristian McDonald, Anna Mikkelsen, Mollie Asbury, Jessica Haver, Hendrikje Jorissen, Marion Chapeau, Robert Toonen, Christopher Suchocki, Van Wishingrad, Christopher Jury, Nina Schiettekatte, Madeleine Hardt, Claire Lewis, Claire Bardin, Joshua Kualani, Crawford Drury, Kira Hughes, Josh Hancock, Carlo Caruso, Andrea Grottoli, Shannon Dixon, Josh Voss, Allison Klein, Sid Verma, Alejandro Alvaro, Richard Argall, Kevin Chun, William Hicks, Alex LeBon, John Yeh, Daniel Wangpraseurt, Samapti Kundu, Lindsey Badder, Stefan Kolle, Erik Franklin, Kelsey Maloney, Guan-Yan Chen

**Affiliations:** 1 Marine Physical Laboratory, Scripps Institution of Oceanography, University of California San Diego, La Jolla, CA USA; 2 Coral Reef Ecophysiology and Engineering Lab, Scripps Institution of Oceanography, University of California San Diego, La Jolla, CA USA; 3 Hawai’i Institute of Marine Biology, The University of Hawai’i at Mānoa, Kāneʻohe, HI USA; 4 Department of Statistics, University of California Berkeley, Berkeley, CA USA; 5 Applied Research Laboratory, University of Hawai’i, 2800 Woodlawn Drive, Suite 263, Honolulu, HI USA; 6 University of Hawai’i at Mānoa, Honolulu, USA; 7 Ohio State University, Columbus, USA; 8 Florida Atlantic University, Boca Raton, USA; 9 Makai Ocean Engineering, Waimanalo, USA

**Keywords:** Bioacoustics, Fish larvae, Acoustic enrichment, Recruitment, Reef restoration, Ecology, Ecology, Ocean sciences, Zoology

## Abstract

Acoustic enrichment (AE)—the playback of ambient sound from healthy coral reefs—shows promise in attracting fish larvae to degraded or artificial reefs, but previous evaluations have used invasive diver-based sampling techniques, limiting most studies to short deployments in benign and accessible environments. This study used fully autonomous cameras to non-invasively evaluate AE efficacy in attracting settlement-stage fish larvae and mature fish over large fractions of a lunar cycle. We deployed an AE system in Kāne’ohe Bay (O‘ahu, Hawai‘i) over three spawning events (August 2023, June–July 2024). Treatment (active speaker) and control (inactive speaker) sites were built by placing artificial structures, autonomous cameras, and hydrophones on a sandy seabed at 5 m water depth, with sites separated by 42–65 m. Treatment and control designations were alternated between deployments to remove potential spatial bias. Manual and semi-automated image analysis found larval counts at both sites peaking around the new moon, but the treatment site attracted 4–14 times more larvae. Both sites encountered similar numbers of mature fish. These results demonstrate that autonomous camera systems can non-invasively study fish larval presence and provide further support that AE can enhance larval fish response.

## Introduction

Fish perform a diverse array of functional processes on coral reefs and play a crucial role in ecosystem rehabilitation^1–3^. Different functional groups of reef fish—including excavators, scrapers, browsers, grazers, and detritivores—contribute to coral reef recovery through distinct ecological processes: excavators and scrapers remove algae and expose bare substrate for coral recruitment, browsers suppress macroalgal canopies, grazers maintain short algal turfs, and detritivores recycle sediment-bound nutrients^4^. The persistence of reef fish populations is largely dependent on the process of recruitment^5^, where juvenile fish, following their planktonic larval phase^6^, utilize various sensory signals to locate and inhabit suitable reef environments^7,8^. Larval settlement and reef health are tightly linked^9^; herbivorous fish recruitment enhances coral recovery by suppressing macroalgae, which is known to inhibit coral settlement^10^. When reefs are in a state of decline, they emit less appealing olfactory and acoustic signals to larval fish, leading to a decrease in fish settlement compared to healthy coral ecosystems^11^. Facilitating the settlement of keystone species such as fish is thus critical for reef resilience and post-disturbance recovery^12^. An emerging technique for supporting accelerated recovery of degraded reefs involves the broadcasting of healthy reef sounds to encourage the recruitment and potential settlement of fish larvae from key functional groups^13,14^.

### Previous research on fish larvae settlement

Settlement, the shift from pelagic to reef-dwelling life stages^7^, is a critical process shaping reef population and community dynamics^15^. Fish larvae do not simply passively drift with currents^16^; they actively select habitats, often showing species-specific preferences that optimize survival^17^. Lunar cycles are key drivers of settlement timing, with larvae regulating development to coincide with specific lunar phases^18^. The mechanisms behind these drivers are both internal and external; for example, specialized photoreceptors detect lunar light cues^19^, while tidal cycles facilitate early-stage larval transport^20^. Species-specific lunar preferences create temporal partitioning in settlement, potentially reducing competition^21^.

Settlement-stage larvae rely on multiple sensory cues: acoustic signals for orientation^22,23^, chemical attractants from corals and microbes^24^, and visual cues for habitat complexity^25^. Physical factors such as substrate structure and hydrodynamics also shape settlement windows^26^. Advances in underwater imaging have improved behavioural observations^27^, but the interplay of multiple cues in natural settings and their application to reef restoration remain active areas of research^28^.

### Acoustic enrichment

**Fig. 1:**
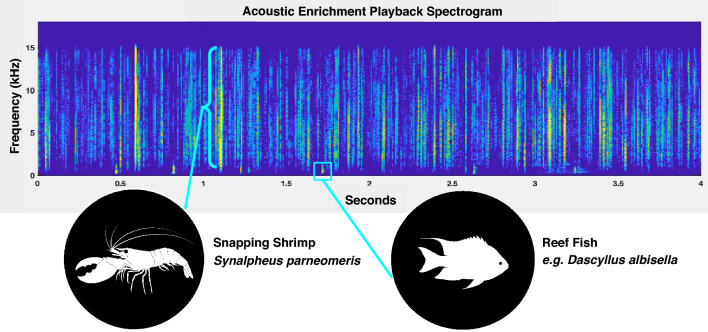
Four-second sample of healthy coral reef sound collected in Kāne’ohe Bay (O‘ahu, Hawai‘i) 300 m from the experimental site during the new moon (August 27, 2022). Hawaiian snapping shrimp (*Synalpheus parneomeris*) and reef fish, such as the Hawaiian Damselfish (*Dascyllus albisella*), dominate the ambient sound field.

Coral reefs produce distinctive underwater acoustic signatures that arise from multiple biological taxa, but particularly from crustaceans (e.g., snapping shrimp) and fish (Figure 1). Reef sounds differ markedly in frequency and amplitude between healthy and degraded reefs, with healthier reefs producing more diverse acoustic environments that are speculated to enhance larval recruitment^29^.

Acoustic enrichment (AE) is the underwater playback of ambient sound recorded from healthy coral reefs at acoustically degraded or artificial reef sites. Settlement-stage fish larvae possess functional acoustic sensors, such as inner ear structures and lateral line systems^30,31^. These enable them to sense acoustic particle motion and pressure over frequency ranges typical of reef ambient sound environments, presumably allowing them to detect and orient toward reef sounds at ecologically relevant distances^7,22,32^.

Previous experimental AE studies using playback of reef sounds have demonstrated significant increases in larval and mature reef fish settlement rates by attracting these organisms to degraded habitats^33,34^. However, these studies relied on daily recovery of settlement tiles and light traps by diver-based surveys, which is invasive and removes larvae from the site, potentially biasing both the behaviour and site persistence of the organisms being sampled^35^. Moreover, light traps themselves introduce artificial illumination that can alter larval distributions, potentially desynchronising settlement timing from natural lunar cues and as a result, attract biased counts of larvae relative to natural conditions^36,37^. The use of light traps and SCUBA also makes AE evaluation difficult in more remote locations, where diver access is limited by depth, weather, distance from infrastructure, or permit constraints. These limitations have helped motivate the development of autonomous, non-invasive monitoring alternatives.

Furthermore, Prior AE studies have not simultaneously analyzed the relative contributions of lunar phase and playback to fish presence; lunar phase is either uncontrolled or treated as a sampling constraint rather than an explicit co-variate in regression models^33,34,38^.

To address both of these challenges, this work builds on recent long-term autonomous AE deployments for coral larvae^39^ by deploying remote autonomous cameras in Kāne’ohe Bay, O’ahu over three lunar cycles (August 2023, June 2024, and July 2024) to simultaneously monitor larval and mature fish settlement in potential response to AE, while also accounting for lunar phase. The open-source cameras use a burst-photo strategy that enables continuous noninvasive and unbiased dawn-to-dusk multi-week observations. They did not require in situ servicing during deployments, allowing larval presence and behaviour to be monitored without additional anthropogenic disturbance beyond AE playbacks, as well as substantially reducing logistical effort.

“Materials and methods” describes the AE equipment, cameras, and fish habitat structures used in the study, as well as the location and experimental layouts. AE data collection and playback schedules are also described, along with manual and automated image review procedures. Finally, evaluation metrics and statistical tests for these metrics are detailed. “Results” then provides the results for larval fish, and Supplemental Materials review the mature fish results.

## Materials and methods

### Overview of study area

**Fig. 2:**
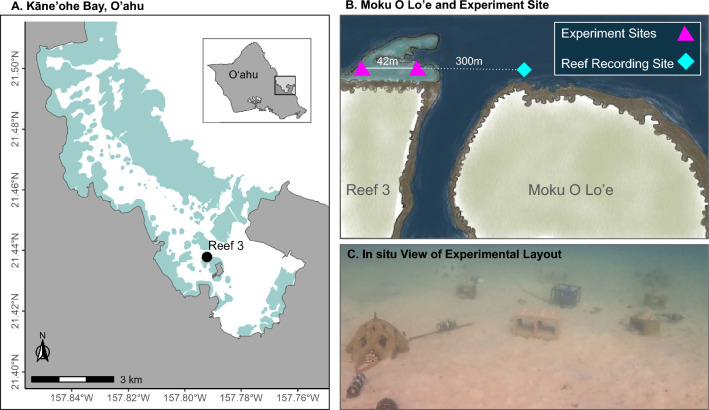
Our field study took place in the Hawaiian Archipelago, off the windward shores of O’ahu. On the northwestern side of Moku o Lo’e Island (**A**) in Kāneohe Bay, Hawai’i. (**B**) provides a diagram of where the control and treatment sites which were located on a sandy flat bottom and 15m from the nearest active reef. The diamond 300 m to the east of the experiment sites represents where the acoustic enrichment “playlist” was collected over the August 2022 new and full lunar phases at a local vibrant reef. Finally (**C**) depicts the in situ arrangement of materials on the sandy substrate, including autonomous camera mountings.

Summer field trials were conducted during fish and coral larval spawning in 2023 and 2024 on the northwestern side of Moku o Lo’e Island in Kāne’ohe Bay, in close vicinity of the Hawai’i Institute of Marine Biology (HIMB). The location had relatively little boat or tourist traffic. Two sites spaced 65 m (2023) and 42 m (2024) apart (approximate locations $21.4378^\circ$ N, $-157.7925^\circ$ W and $21.4378^\circ$, $-157.7919^\circ$) were located on a flat sandy bottom (Fig. 2D) at a depth of approximately 4–5 m, depending on the tide level. Both sites were approximately 15 m from any nearby natural fringe reef outcroppings, and both had identical visual setups, including identical playback speakers, to ensure consistent visual cues between the locations. During a specific deployment one site served as a control location with an inactive speaker, while the other site received acoustic enrichment from an activated speaker. The location of the control and treatment sites were swapped between deployments to counter potential site bias in recruitment activity. For instance, in June 2024, the treatment site was the eastern location, while in July 2024, it was at the western location.

### Equipment

#### Acoustic enrichment (AE) equipment

The design and implementation of an autonomous underwater acoustic playback system without surface expression was centered around a programmable PIC-32 microcontroller. The system is powered by 180 D-cell NiMH batteries to achieve an energy storage capacity of 3.8 kW-hr, sufficient for three weeks of continuous nighttime operation without the need for recharging by solar panels or shore-based cables. The batteries are divided between two pressure cases with a length of 81.3 cm and diameter of 21.6 cm, which are deployed on the ocean floor side by side, with a 10 m cable connecting a Lubell LL916C underwater electrodynamic loudspeaker to one case. Another 10 m cable connected the two cases, permitting each pressure case to be lowered independently from a boat without requiring connections to be made underwater. An identical silent speaker was deployed at the control site. At the treatment site the gray pressure cases were placed 10 m to the west of the speaker to reduce the odds of the cases visually attracting animals.

The system features a magnetic switch that allows for in-situ testing of the playback functionality. This switch activates a six-minute playback test, independent of any pre-set scheduling, providing a flexible and reliable testing framework to confirm playback. The system software has a unique “playlist” for each day of the month, with each playlist listing the names of multiple WAV-formatted files. This feature is important as the ambient sound of a reef may vary subtly with the phase of the moon. The software has a master schedule specifying start and stop times each day of the month. Whenever playback begins each evening, the playback device broadcasts each file in the playlist sequentially, after playing an initial six-minute test file. An internal log notes the specific file and time activated. A SoundTrap ST 300 (Ocean Instruments, NZ; sensitivity 173.3 dB re 1 $\upmu$Pa/V), an autonomous digital hydrophone recorder, was placed 1 m from the speaker to confirm and characterize the playbacks. The ST 300 was configured with a 96 kHz sample rate on a 50$\%$ percent duty cycle (30 min on / 30 min off) to extend deployment endurance to 21 days using the internal lithium battery pack.

#### Fish habitat structures

Across three deployments, two different combinations of artificial reef structures were deployed as fish larvae habitats: “Stacks” (3 modular tubes ($20 \times 20 \times 20$ cm)) and “Fish Habitat Modules” (FHM) (50 $\times$ 25 cm (diameter $\times$ height)), which contain hexagon-edged tunnels intended to provide fish habitat^40^. The last two deployments also included smaller “Coral settlement modules” (CSM) (20 $\times$ 10 cm (diameter $\times$ height)), which were deployed around the treatment and control sites to evaluate coral larvae settlement as part of a separate experiment^41^. The FHM and CSM designs were constructed in Blender v3.4. and were either printed in clay or concrete casted in a silicone mold. The printing in clay used mid-fire pottery clay (Soldate 60, Aardvark Clay and Supplies, United States) and printed with the Delta Wasp 40100 3D clay printer (Wasp, Italy), air-dried and fired in a kiln (Jupiter Sectional Kiln, L&L Kilns, United States). When casted in concrete, a silicone mold was used; the mold was produced from a 3D plastic print (BigRep One 4, Germany).

The initial 2023 deployment also used a simple rubble pile, which consisted of calcareous rock pieces, $\approx$ 5–15 cm diameter, arranged in an irregular mound of $\approx$ 30 $\times$ 15 cm (diameter $\times$ height).

Finally, during the last two deployments bundles of 13.3 cm long PVC pipe were bound together to mimic standardized larval fish habitat designs, known as “fish-specific autonomous reef monitoring structures (FARMS)”^42^. Each bundle used five 1.25 cm diameter pipes and five 1.9 cm diameter pipes. These PVC bundles were sometimes placed within a FHM or placed separately.

**Fig. 3:**
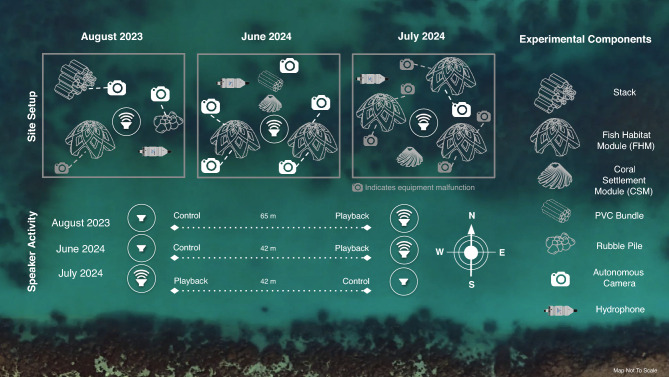
Detailed layout of structures and cameras at treatment and control sites for each deployment. Although the exact number and kinds of structures changed between deployments, for any given deployment, the numbers, placement, and types of structures were identical between the treatment and control sites.

While the exact combinations of fish habitats varied between deployments, the exact same sets of structures were placed at both the treatment and control sites for each deployment, and all structures were placed 2 m from the speaker (Fig. 3):

- First deployment (August 2023): a stack was deployed alongside a single FHM and rubble pile at each site.
- Second deployment (June 2024): two FHMs (each with a smaller PVC bundle), two CSMs and an independently placed PVC bundle were deployed at each site.
- Third deployment (July 2024): three FHMs (each with a smaller PVC bundle) and two CSMs were deployed at each site. Two PVC bundles were inserted inside each FHM, but only one camera survived from each site.

#### Autonomous cameras

For continuous visual monitoring, an open-source camera system was employed based on the ESP32-S chip and an OV2640 camera (2MP 1600 $\times$ 1200 resolution) with a 65-degree field of view (FoV). In 2024 deployments, one camera used an adapter that permitted a 160-degree FoV. A custom real time clock circuit board (Ecologis Consulting, Inc.) used an Arduino microcontroller to act as a scheduler and intervalometer for activating the ESP32-CAM image acquisition.

The open source software was modified to permit burst-mode functionality, which allows the capture of images in rapid succession to effectively detect and document motion. The data acquisition is completely silent and non-invasive. The cameras did not use external lighting, and thus only operated during daylight hours and automatically adjusted its sensitivity based on lighting levels. Because no AE took place during the day, the use of the cameras assumes that any larvae attracted to the site during the night will persist at least into the following morning. Prior literature suggests that settlement-stage larvae of many coral reef species are known to shelter at or near settlement structures during daylight hours following nocturnal arrival^25,27^, supporting this assumption. We acknowledge, however, that early post-settlement mortality and active daytime dispersal could cause our counts to underestimate total overnight larval visitation.

Two packs of (6) AA 2400mAh 7.2V NiMH batteries powered both the ESP32-CAM and cameras, with everything encased in a 5 cm diameter, 15 cm long acrylic pressure case (Blue Robotics, Inc). The cameras permitted continuous daylight coverage, averaging 13 hours a day for up to 21 days without replacement.

The image sampling protocol differed slightly between the 2023 and 2024 experiments. In 2023, the cameras were programmed to acquire five sequential images (taken over $\approx$ two seconds) at 120 second intervals. However in 2024, the sampling frequency was increased to 300 seconds to extend the camera deployment operation to over 21 days and match the duration of the acoustic playback protocol.

In 2023 the cameras were tie-wrapped to a 2 kg lead weight and placed roughly 30 cm from a given structure (stack, rubble, or FHM) directly on the sandy bottom. In 2024 two cameras were assigned to each FHM. Each camera was mounted on a 1.27 cm diameter threaded steel rod 33 cm away from a FHM entrance, ensuring consistent distance and coverage for each camera (Fig. 2D). All structure-facing cameras were fitted with the same 65-degree FoV.

An additional camera attached to a 2 kg lead weight was placed on the sea floor with the 160-degree wide-angle lens to capture both the standalone fish stack along with the entire site. The wide-angle camera was used exclusively to provide a site-wide view for qualitative assessment and was not included in the quantitative MaxN or CC calculations reported in the results, which are based solely on the $65^\circ$ cameras mounted on standardised rods at identical distances from each FHM entrance.

### Summary of deployment layouts and camera coverage

Figure 3 displays an aerial layout of the complete experimental set across all three deployments. The 2023 deployment shows the relative location of the speaker, cameras, and habitats (stacks, rubble, and FHM). The site layout evolved in June and July 2024, which included the addition of more cameras and the replacement of the stack and rubble piles with additional FHMs circling the speaker. Each FHM incorporated 1-2 PVC bundles. Additionally, CSMs were deployed to assess the effectiveness of acoustic enrichment for coral larvae recruitment for a separate study^41^. Although the exact number and kinds of structures changed between deployments, for a given deployment the numbers, placement, and types of structures were identical between the treatment and control sites.

The number of working cameras varied by deployment, illustrated in Fig. 3. The August 2023 deployment had three cameras per site. One camera at the control site failed between 8/15 and 8/21, thus that data has been excluded from the treatment site during those same dates when computing statistics. During June 2024, ten cameras were successfully deployed with endurance between 14 and 22 days, with two cameras assigned to each of the FHMs, and a wide-angle camera each assigned to a control and treatment site. In July 2024 twelve cameras were deployed, with two assigned to each of FHMs, plus a wide-angle camera assigned to control and treatment sites.

Figure 3 also illustrates the number of surviving and malfunctioning cameras from the three deployments. The worst failures occurred during the last July 2024 deployment, when all but three cameras malfunctioned almost immediately, even though the cameras had worked well the previous deployment in June 2024. Fortunately, the surviving cameras were split between the control and treatment site and recorded for 11 and 12 days each, respectively. One of the treatment cameras was angled down toward the sand, providing an obstructed view of the fish habitats, and thus only one treatment and one control camera was used for analysis in July 2024.

### AE data collection and playback

Passive acoustic data used by the playback device was collected by a SoundTrap ST 300 at $21^{\circ }$26’15”N $157^{\circ }$47’25”W in Kāne’ohe Bay during August 2022, a location 300 m from the eventual test site (Fig. 1). The recording site ($21^\circ$26$'$15$''$N, $157^\circ$47$'$25$''$W) has been surveyed by HIMB snorkelers for years, and was chosen because it had substantial and diverse live coral cover, little fleshy algae, supported varied fish assemblages, and contained snapping shrimp (*Alpheus* spp.) and damselfish (*Dascyllus albisella*). The presence of these organisms was detected by the SoundTrap during recording, which are both known to be dominant contributors to Hawaiian reef soundscapes^43^. We use the term ‘healthy’ in the sense of prior work^29^ to denote a reef with above-average live coral cover and richer biological sound production relative to the experimental site.

The SoundTrap continuously recorded healthy coral reef sound at 96 kHz sampling rate for eight days centered around the full moon phase, and an additional eight days centered around the new moon phase. These data were decimated (low-pass filtered then down-sampled) to 36 kHz WAV files using an FIR lowpass filter, trimmed to begin at sunset and end at sunrise, and transferred to the 128 Gb flash memory on the playback equipment.

Acoustic playbacks were scheduled daily to broadcast continuously from 17:30 to 7:00, with most days featuring unique data and no repetition. This dusk-to-dawn playback timing mirrored previous successful AE demonstrations^34^, and was both a pragmatic and ecologically grounded choice. Reef sounds are naturally potently pronounced during evening and nocturnal periods coincide with peak vulnerability and settlement activity of many larval fishes^44^.

The files chosen from 2022 were assigned to be broadcast on days that matched the same lunar phase as the data of the original recording. If a particular playback day had no corresponding recorded data from a corresponding lunar cycle, a recording day with the closest match was used, so as a result some playback recording days were repeated with the same playlist.

**Fig. 4:**
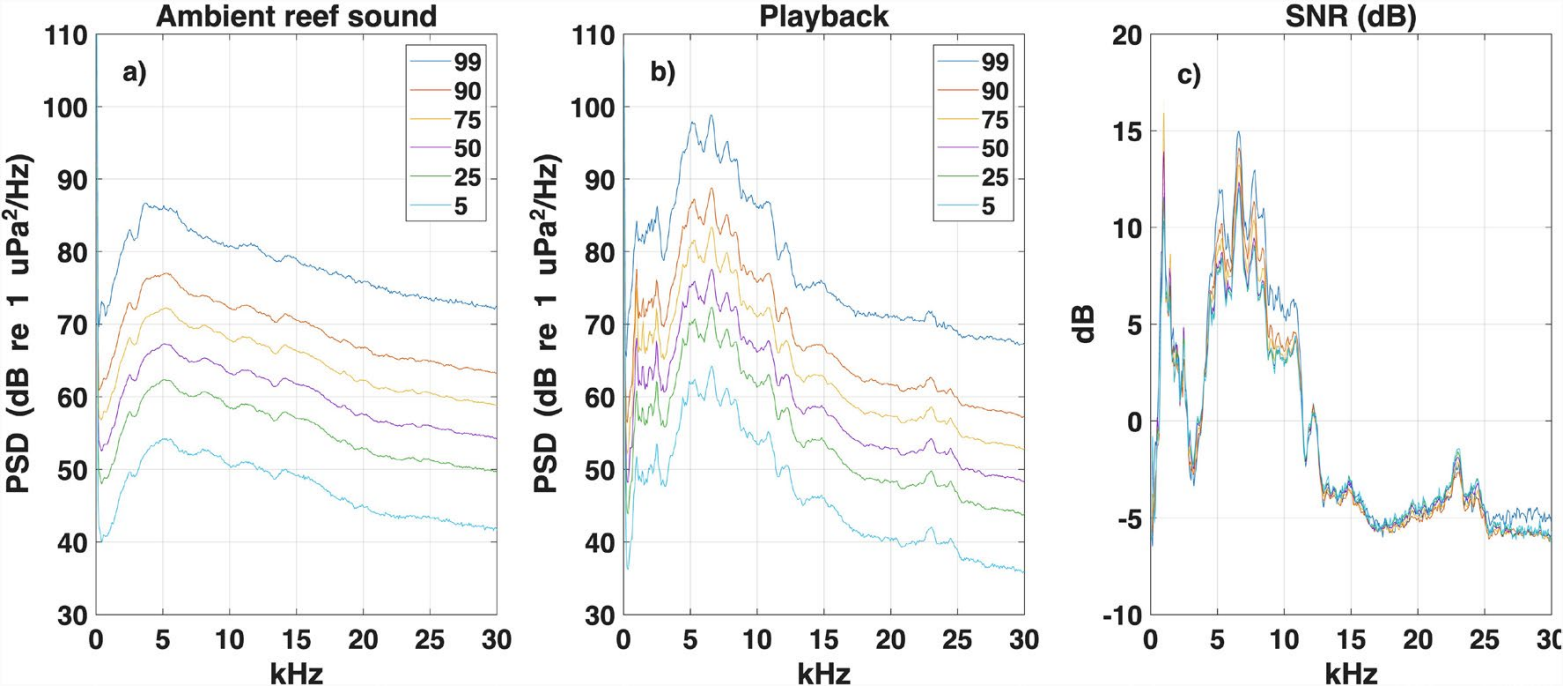
Estimated power spectral density levels (dB re 1 $\mu \text {Pa}^2$/Hz) at 2 m range from speaker, using data collected at 1 m range and assuming spherical spreading. Each colored line represents a percentile. (**a**) Background level percentiles measured at 17:11 local time on July 5th, 2024 (new moon); (**b**) levels after playback began at 17:30; (**c**) difference in levels (signal-to-noise ratio) in dB.

The Lubell speaker was placed so that its transmission main lobe (peak directivity) was pointed vertically, for two reasons. First, it ensured that the sound transmission was azimuthally symmetric, and it also ensured that sound projection was focused toward the ocean surface. An engineering deployment in July 2023 placed the SoundTrap at ranges of 6, 12, 24, and 48 m from the active speakers. The resulting measurements were used to adjust the playback source level so that the root mean square (RMS) received levels (computed between 1 and 15 kHz) matched ambient RMS levels (SNR 0 dB) at 20 m range from the treatment speaker, or roughly half the distance to the control speaker. Power spectral density (PSD) levels for the final playbacks were measured at 1 m range using the same SoundTrap that collected the original data. Figure 4a displays percentiles for natural background PSD levels before playback begins, and subplot (b) displays estimated received PSD levels at 2 m range, the distance of the settlement structures from the speaker. The estimated levels were obtained by subtracting 6 dB from the measured levels at 1 m range, assuming spherical spreading. The final subplot displays the signal to noise ratio (SNR) between the playback at 2 m range and background noise levels as a function of frequency.

**Fig. 5:**
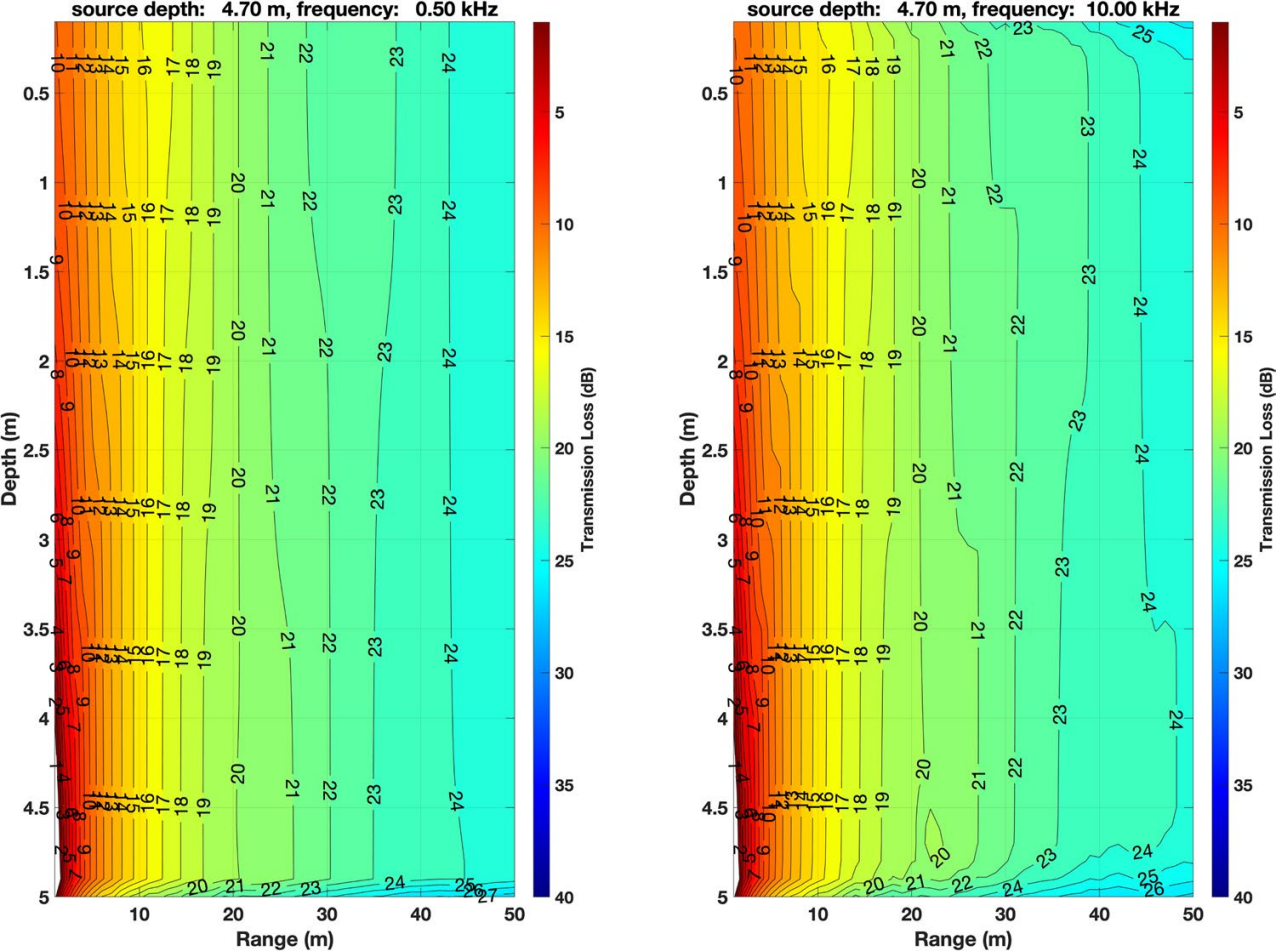
Modeled propagation loss (dB) from directional Lubell speaker. Contour plots of loss are shown for 500 Hz (left) and 10 kHz (right) frequency.

Acoustic modeling was used to estimate the propagation range of the acoustic playback versus frequency (Fig. 5). The flat bathymetry significantly simplified acoustic modeling of propagation loss, using the incoherent version of the ray-tracing program BELLHOP^45^. Inputs to the model include the directional properties of the Lubell speaker^46^, a water depth of 5 m, and bottom properties associated with limestone (3000 m/s compressional speed; 2.4 $\text {g/cm}^3$ density). Although the seabed interface was composed of white sand, simulations using a deep sand bottom yielded higher propagation losses with range than measured with the July 2023 field measurements, suggesting that the sand is only a thin layer over a larger coral or limestone-comprised basement. Figure 5 shows a contour map of the predicted propagation loss for both 500 Hz and 10 kHz. The propagation loss varies little with depth, except for higher losses a few cm above the ocean floor. A 15-20 dB SNR measured in the data at one meter suggests that the acoustic enrichment broadcast at 15 dB SNR (Fig. 4c) would disappear into the background noise at ranges where the propagation loss was between 16 and 21 dB, which corresponds to 11–26 m range from the speaker. The 5 dB SNR of the ultrasonic component is predicted to merge into the ambient background at only 2–3 m range. Simulations at lower frequencies (100 Hz) showed similar loss characteristics as the 500 Hz results shown here.

Acoustic modeling also showed that the acoustic particle velocity of the broadcast was typically proportional to the pressure field at ranges more than a few wavelengths for the source, except within a half a wavelength of a reflecting boundary (e.g. within several cm of the ocean floor). These conclusions are consistent with experimental guidance^47^ and recent high-quality field measurements^48^. As a result, independent particle velocity measurements were not required for this site, and the 21 m propagation range estimated here is consistent for both pressure and particle velocity.

Fieldwork involved passive acoustic monitoring via autonomous cameras and acoustic enrichment via underwater speaker playback of ambient coral reef recordings. For this experiment no formal approval by University of California San Diego’s Institutional Animal Care and Use Committee (UCSD IACUC) was required because this study was purely observational in nature and did not involve capture, handling, invasive procedures, or interventions expected to materially alter the biology, behavior, or ecology of the study animals. No animals were captured, handled, confined, or harmed at any point during the study. Acoustic playback levels were calibrated so that received sound levels matched ambient reef noise levels at the scale of the experimental site. This determination was provided by UCSD IACUC in consultation with the National Institutes of Health (NIH) Office of Laboratory Animal Welfare (OLAW) field research guidelines (NIH/OLAW FAQ on Field Research; Guide for the Care and Use of Laboratory Animals, 8th ed., Appendix A). All fieldwork was conducted in compliance with applicable local, state, national, and international wildlife regulations.

### Image review procedures

As mentioned previously, the image analysis was only conducted on cameras with a $65^\circ$ FoV. The analysis took place over two stages: an initial manual review of the burst images, followed by an automated analysis in circumstances where the number of larvae in an image were too great for reliable manual counting. Despite a limited 2MP resolution in the ESP-32 cameras, the presence of fish larvae could be identified by the initial manual review, which distinguished larvae from passively drifting flotsam in the water by noting differences in motion over multiple burst images. Flotsam typically moves in linear trajectories with no acceleration, while and fish larvae moved in nonlinear trajectories with acceleration, creating a “swarming” motion.

During the manual review each set of burst photos was flagged if larvae were detected, ensuring that only images containing potential targets advanced to the automated analysis stage. If individual organs (e.g. a yolk sac) were visible in the image, the organism was considered, and counted, to be in the larval phase. All mature fish camera presence was manually marked.

In the 2024 deployments, the number of larvae present in any given image was sufficiently small that the datasets could be analyzed manually. Three reviewers visually assessed all image data, with larvae-abundant images reviewed independently by two reviewers to confirm consistency of the results.

However, during 2023, the initial year of the study, the number of larvae visible in the images was large enough that bulk manual counts were impractical. A simple image segmentation algorithm was developed to count larvae in photo sequences that were flagged as having larvae (resulting figure located in Supplemental Materials). The algorithm subtracted two successive burst images from each other, highlighting objects that moved between frames. After applying a threshold to convert the output into a binary image, a morphological opening operation was used to restrict counts to objects containing between 5 and 100 pixels. The number of detected objects was divided by two to yield the final larval count, accounting for the fact that a moving object produces two objects in the subtracted image. All segmentation outputs were subsequently reviewed manually to ensure that detections were consistent with annotation standards established during the 2024 dataset, providing an additional layer of validation between the automated approach and ground-truth labeling. A formal manual versus automated comparison on a subset of frames is provided via an interactive web page accessible from the Supplemental Materials.

### Data analysis

#### Metrics

Raw counts of mature fish and fish larvae in individual photographs are translated into three metrics to compare between the treatment and control site for each deployment. The metrics are evaluated over both 6-h and 24-h time intervals during daylight hours to create time series, as well as across the entire deployment.

- Counts per camera (CC): the raw number of fish larvae visible across all photos from all cameras at a site over a time interval, divided by the number of functioning cameras. It is defined as follows:
  1 $$\begin{aligned} CC_i = \sum _{k=1}^{N_c} \sum _{t\in T_i} N_k(t)/N_c \end{aligned}$$
  where $T_i$ represents the $i_{th}$ time interval, $N_k(t)$ is the fish or larvae count on camera *k* at time $t$, and $N_c$ is the total number of cameras available during the time interval. The CC metric has the advantage of being intuitive to understand and of maximizing detection probability of larvae, particularly in low-density environments where rare individuals may be missed by more conservative approaches. However, it has the disadvantage of blending individual counts with fish persistence.
- MaxN: the maximum number of individuals detected in a single image across a time interval. It is defined as follows:
  2 $$\begin{aligned} MaxN_i = \max _{t\in T_i, N_c} N(t) \end{aligned}$$
  MaxN is considered the metric most closely related to the true population of individuals in an area; it is widely used in marine ecology because it minimizes double-counting and standardizes comparisons among samples^49,50^. If multiple cameras are operating simultaneously at a given site (treatment or control), then MaxN is redefined as the maximum number of individuals visible in a single image across all camera images at that site within the time interval. The reason behind this definition is that the cameras are not time-synchronized, so there is a small probability of double-counting larvae in contemporary photos taken from two nearby cameras.
- Time fraction: the fraction of images collected over a time interval that contain images of fish larvae and/or mature fish, which can be interpreted as the fraction of time fish are visible during the time interval. This metric provides a guide to how persistent fish presence is at a location over a time interval.

#### Statistical analysis

We conducted a non-parametric Wilcoxon rank-sum test in R to evaluate statistical difference in the medians between the treatment and control site, and then fit a parametric generalized linear model to estimate the effect size.

We assume that the statistical process generating the metrics are ergodic: that time series samples are independent and representative samples of the fundamental statistical process. Autocorrelations of the MaxN and CC time series were used to select a time window that yielded uncorrelated sequential samples, which was determined to be 24 h. We then applied the Wilcoxon rank-sum test between the treatment and control site for each deployment, for the CC, MaxN and Time fraction metrics. This test makes no assumptions about the form of the underlying generating distribution, but tests the null hypothesis that the samples arise from the same underlying distribution. We restricted the time samples to be within 4 days of the new moon (8–9 samples), to standardize the time coverage between deployments, and to exclude dates long after the new moon when no larvae were present at either site.

If a potential difference between sites is flagged, we estimated the effect size by applying a generalized linear model^51^ to the MaxN metric, which we assume can be parametrically modeled by a zero-inflated Poisson (ZIP) process, since the sample data were non-zero integer counts with significant time periods when no larvae or fish were observed. This analysis is applied to the MaxN metric only, because it is assumed to be the best proxy for actual population size. Under this model, the probability of detecting none of object type *h* (larvae or mature fish) at site *j* is as follows:

3 $$\begin{aligned} P_{hj}(n_{hj}=0) = \pi _{hj} + (1-\pi _{hj})e^{-\lambda _{hj}} \end{aligned}$$

and the probability of detecting $n_{hj}$ individuals of type *h* in a photo at site *j* becomes

4 $$\begin{aligned} P(n=n_{hj}) = (1-\pi _{hj})\frac{\lambda _{hj}^{n_{hj}} e^{-\lambda _{hj}}}{n_{hj}!} \end{aligned}$$

where $\pi _{hj}$ is the probability of generating a sample from a pure zero process for object *h* at site *j*, and $\lambda _{hj}$ is the expected mean for the Poisson process.

$\lambda$ was modeled as linear regression on two independent variables and an intercept value, using a log link function^51^. The independent variables were (1) a categorical variable *TREATMENT*, indicating whether the data were taken from the treatment or control site, and (2) a continuous variable *MoonPhase*, which represents the phase of the moon when a sample is taken, expressed in terms the fraction of the moon’s surface illuminated. A new moon is thus represented by 0, and the full moon by 1, with both waning and waxing phases represented by the same number.

The regression formula for the mean MaxN value for object type *h* becomes

5 $$\begin{aligned} \ln [\lambda _{h}] \sim {b_{h,0}+b_{\textit{h,MoonPhase}}\cdot {{\bf MoonPhase}} + b_{h,treatment} \cdot {{\bf TREATMENT}}} \end{aligned}$$

with $b_{h,0}$, $b_{\textit{h,MoonPhase}}$, and $b_{h,treatment}$ being the regression coefficients for the intercept, moon phase, and treatment site respectively. As with the Wilcoxon rank-sum test, time samples were restricted to be within 4 days of the new moon. A separate regression was conducted for each of the three deployments.

Assuming a non-informative prior, the regressions were solved using the Bayesian generalized multivariate model *brms* package^52^ in the statistical software package R^53^. The package provides an interface for fitting Bayesian regression models using the probabilistic programming language Stan, which in turn uses Hamiltonian Monte Carlo (HMC) sampling algorithms to solve multidimensional integrals^54^. Each regression ran four chains with 4000 iterations each, of which 2000 were used for the “warm up” phase, using a target average acceptance probability (*adaptive_delta*) of 0.95. Models with interactive terms were also tested, and the Leave-One-Out Cross-Validation (LOOCV) metric^55^ evaluated whether to include these additional terms in the model.

Coefficients with 95% credible intervals that excluded zero were interpreted as indicating a statistically significant effect of that factor on MaxN. The effect size is provided by the expected ratio of MaxN between the treatment and control sites, provided by the expression $e^{b_{h,treatment}}$.

## Results

### Acoustic playback shows increased larval fish presence

This section presents results for fish larvae only. The equivalent analyses for mature fish are contained in the Supplemental materials.

Table 1 lists the MaxN, CC, and Time Fraction metrics computed over the entire deployment time window, for each of the three deployments at the eastern and western sites. Purple shaded areas indicate the treatment site. The locations of the playback and treatment site were swapped between June and July 2024 (Fig. 3), yet Table 1 clearly shows more fish larvae were always detected at the treatment location (highlighted in purple) across the entire deployment, measured in terms of any metric, regardless of whether the treatment site was placed in the western or eastern location. This suggests that site-specific geographical factors are not responsible for the elevated counts at the treatment site.

**Fig. 6:**
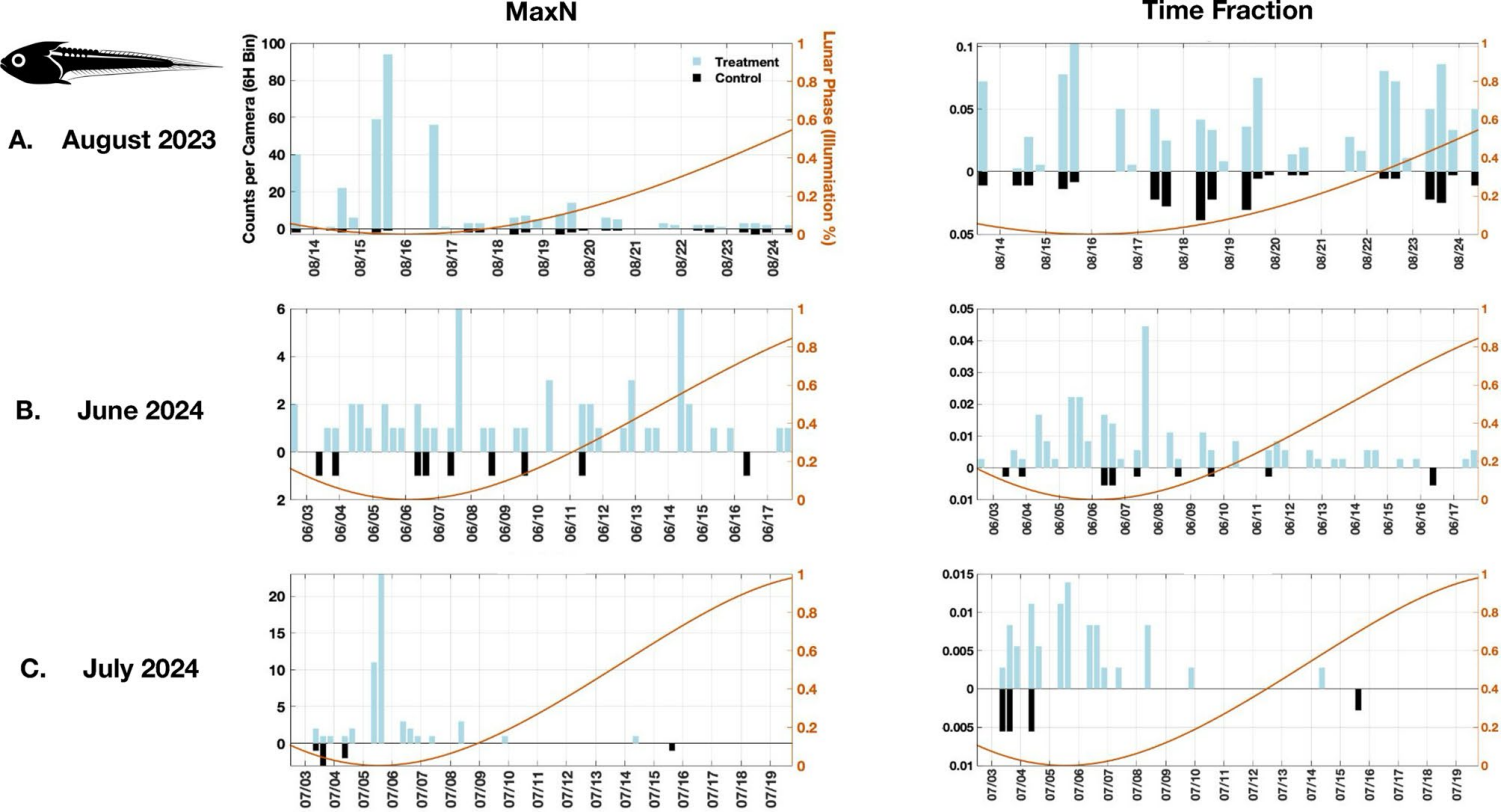
Time series of MaxN (left column) and time fraction (right column) metrics, evaluated every six hours during the three deployments at the treatment (blue) and control (black) sites, with control values reflected about zero for display purposes. Lunar phase (right y-axis) is displayed as an orange line. The deployment months were, respectively (**A**) August 2023, (**B**) June 2024 and (**C**) July 2024.

**Fig. 7:**
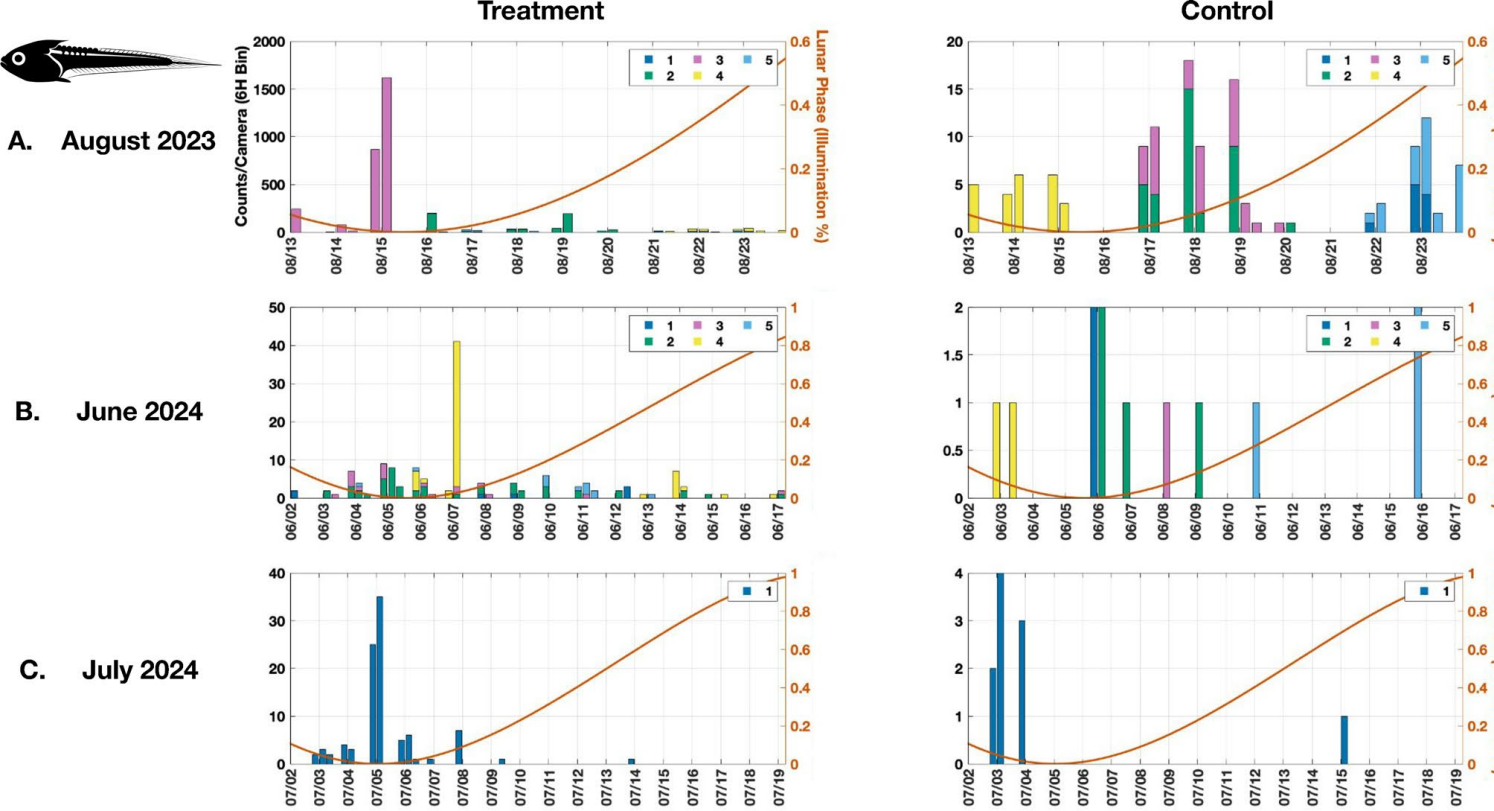
Fish larvae counts per camera for (**A**) August 2023, (**B**) June 2024, and (**C**) July 2024 deployments, computed every six hours, with different cameras represented by unique colors. Left column shows treatment site; right column shows control site. Lunar phase (right vertical scale) is displayed as an orange line. Note: *y*-axis scales differ between treatment and control panels to aid visibility of low-count control data.

Figure 6 displays time series of the MaxN and time fraction metrics for all three deployments, computed over six-hour intervals. This figure illustrates temporal variations in both larval abundance (MaxN) and presence (time fraction), with clear dependencies on lunar phase. In August 2023, larval counts and presence were sharply elevated immediately following the new moon, peaking within a narrow window before rapidly declining (Fig. 6A). In June and July 2024 (Figs. 6B, C), larval detections were much lower overall and more evenly distributed over time, but again highest close to the new moon, particularly at the treatment sites. Figure 7 displays time series of the raw counts for each camera, with individual colors representing different cameras, illustrating that differences in raw counts are detected across all working cameras at a site, with temporal patterns similar to the MaxN results.

Table 2 summarizes the Wilcoxon rank-sum hypothesis tests for the CC, MaxN and Time Fraction metrics across all deployments, using 24 hour time windows between samples, and restricting samples to those within 4 days of the new moon. All tests show statistically significant differences between the sites ($p < 0.05$ for all), although the low number of cameras available during July 2024 makes the result marginal. The table also shows the coefficient estimates of the ZIP regression model for MaxN, with “AE Multiplier” showing the ratio of $\lambda$ between the treatment and control site. Estimated mean MaxN rates were consistently larger at treatment sites; across the three deployments (Table 2) the AE multiplier effect was between 4 and 14. The regression analysis computation excluding lunar phase has changed the values of $b_{treatment}$ by less than 1%. The LOOCV analysis found interactive terms to be insignificant. In other words, lunar phase did not interact significantly with treatment site.

The Wilcoxon rank-sum tests for fish larvae all yield statistically significant differences between the treatment and control sites across all three deployments, for all metrics. The subsequent ZIP regression modeling finds $b_{treatment}$ yielding 95% confidence intervals (CI) that exclude zero, and is thus a significant factor in predicting the MaxN metric. The moon phase effect $b_{MoonPhase}$ is also found to be significant for all deployments except June 2024, where the CI span zero. The moon phase coefficient is always negative, indicating that MaxN counts decrease during nights further from the new moon, while the positive values for the treatment coefficient indicate that MaxN counts are greater at the treatment site.

Once lunar phase effects are accounted for, the resulting effect sizes (AE multiplier effect) are 4 to 14 times greater at the treatment vs. the control site, depending on the deployment, with the multiplier effect between June and July 2024 being quite similar (4.1 vs 4.4). The 95% confidence intervals for $b_{treatment}$ always yield a multiplier greater than one, regardless of the deployment. These AE impacts seem substantially higher than those reported in previous reef-sound light-trap work, whose multiplier effects range from 1.67^33^ for early-stage fish larvae in initial light-trap studies and up to twice the abundance during multi-week patch-reef playback experiments for juveniles^34^.

The Supplemental materials display similar analyses for mature fish presence, but found no significant difference in presence between the treatment and control sites. Examples of frequent organisms sighted by our autonomous cameras at both sites include ringtail surgeonfish (*Acanthurus blochii*), yellowfin surgeonfish (*Acanthurus xanthopterus*), conspicuous sea cucumbers (*Stichopus conspicuous*), green sea turtles (*Chelonia mydas*), Hawaiian trevally (*Caranx melampygus*), Hawaiian goby (*Sicyopterus stimpsoni*), and their symbiotic partner, the Hawaiian snapping or pistol shrimp (*Alpheus* spp.).

**Table 1:** Fish larvae metrics computed at eastern and western sites during three deployments. “MaxN” is the largest number of larvae seen in a single photograph within a 5-image burst sequence, “CC” is the average number of larvae observed per camera across the entire deployment, and “TF” is the time fraction across the deployment. August 2023 numbers are a manually-counted subsample of all days. Bold highlights indicate the treatment sites. Cameras used in the analysis are detailed in “Summary of deployment layouts and camera coverage”.

Deployment	Cameras	MaxN (East )	MaxN (West )	CC (East)	CC (West)	TF (East)	TF (West)
August 2023	4	5	**81**	0.71	**6.52**	5.7e-3	**2.3e-2**
June 2024	10	1	**6**	2.6	**33.8**	3.9e-4	**3.7e-3**
July 2024	2	**29**	3	**112**	37	**1.2e-3**	2.6e-4

**Table 2:** Summary of statistical tests for fish larvae during all three deployments, restricted to times within 4 days of the new moon. Results in bold indicate statistically significant (p < 0.05) differences between treatment and control sites for the rank-sum test, or regression coefficients whose 95% CI excludes zero. The regression coefficients are defined in Eq. (5), and the AE multiplier $e^{b_{treatment}}$ is the estimated ratio of mean MaxN between treatment and control sites.

Month year (samples)	Wilcoxon rank-sum test		Zero inflated Poisson regression			
	(rank sum/p-value)			(Estimate [95% CI])		AE multiplier
	Counts/camera	MaxN	Time fraction	$b_{treament}$	$b_{MoonPhase}$	
Aug 2023 (8)	*40/* ***8.0e-3***	*99/* ***4.6e-4***	*93/* ***6.8e-3***	**2.63** [2.10 3.24]	**-17.93** [-22.21 -13.79]	13.9
June 2024 (9)	*125/* ***8.2e-5***	*118.5/* ***2.3e-3***	*123.5/* ***2.0e-4***	**1.41** [0.42 2.53]	**-2.20** [-8.75 3.86]	4.1
July 2024 (8)	*91/* ***8.0e-3***	*87/* ***0.04***	*90/* ***0.016***	**1.48** [0.26 2.76]	**-26.88** [-40.55 -14.70]	4.4

## Discussion

Our proposed explanation for the higher AE impacts measured in this study is that our autonomous, long-duration monitoring design permitted us to avoid behavioural biases introduced by diver servicing and artificial illumination, and was able to detect transient reef-arrival and visitation events with less bias than daily serviced light traps. The cameras may also have been able to detect more larvae before substantial mortality or dispersal set in, when compared with other time-delayed sampling methods.

While AE treatments consistently attracted significantly higher numbers of fish larvae compared to control sites, no significant differences in mature fish presence was noted at either site (see Table 1 in Supplementary Materials). We believe this finding arises due to non-territorial mature fish in this region having natural foraging areas with linear dimensions of tens of meters^56^, which can well exceed the 42-65 m separation between the treatment and control site.

Our camera measurements also found that fish larvae presence generally dropped off substantially within a couple days of the new moon, a relatively short visitation time compared to other AE studies^34^. Our ZIP statistical regression found that lunar phase was a significant predictive factor for larval presence, in that $b_{MoonPhase}$ 95 % CI excluded zero for two out of the three deployments, thus providing verification for the qualitative observations that larval presence was highest around or at the new moon. However, the effect size of including lunar phase on the AE results were insignificant; when lunar phase was excluded from the regression, the resulting change in the AE coefficients changed by only $\approx$ 1%. By restricting the statistical analysis to a 4 day window around the new moon, we likely lowered the impact of moon phase in the statistical regression. This difference may arise from natural dispersive behaviour or mortality of larvae after visitation, or perhaps because the scale of the artificial structures was too exposed, or otherwise insufficient for the settling larvae^57^, forcing them to disperse after a couple of days^58,59^. Thus, while our results demonstrate that AE can enhance larval visitation, it remains uncertain whether settlement structures also need to provide adequate habitat and shelter to retain larvae and support long-term recruitment. This question remains an important avenue for future investigation.

Point-source playback attenuates more rapidly than the diffuse, spatially extended soundscape of a healthy reef, and field measurements confirm that speaker-radiated sound is indistinguishable from ambient background noise at distances as short as 50 m^34^. This propagation characteristic necessarily restricts the effective acoustic detection range available to approaching larvae, and may have caused the 4–14$\times$ effect we observed to be a conservative underestimate of what a spatially distributed soundscape would produce.

Prior work has shown that larval responses are distance-dependent, attenuating with increasing distance from the speaker^23,34^, and that spatially variable soundscapes produce spatially variable larval settlement^60,61^. An array of synchronised speakers distributed across the settlement structure, approximating the spatially coherent and geographically extended sound field of a natural reef, would more honestly replicate authentic reef acoustics and could further increase both the detection range and the magnitude of the larval fish attraction effect. We therefore regard point-source playback as a conservative implementation of acoustic enrichment, and recommend that future restoration deployments explore spatially distributed speaker arrays as a means of extending the acoustic footprint of enriched structures.

During 2023, the deployment recorded roughly two orders of magnitude more larvae (by Camera Counts (CC); see Fig. 7) than either 2024 deployment, and the underlying cause remains unclear. Perhaps larval spawning is generally greater later in the spawning season. However, informal discussions with Hawai’i Institute of Marine Biology stationed researchers whom work in the bay suggest that spawning activity in 2023 was unusually high across both fish and corals relative to subsequent years. This discrepancy illustrates the importance of uncontrolled factors such as seasonal timing and annual environmental conditions on absolute larval settlement counts. However, our control/treatment experimental design allowed us to discriminate AE effects from other large effects like timing within a spawning season, or environmental differences between seasons.

### Future applications and challenges of acoustic enrichment

Acoustic enrichment is a promising future tool for reef restoration and enhancing fish recruitment across diverse coral reef ecosystems globally. By broadcasting recordings of healthy reef soundscapes, AE can attract settlement-stage larvae to degraded habitats, potentially accelerating ecosystem recovery and biodiversity replenishment. However, our study finds that the technique is best suited for attracting fish larvae, and not fish in other life-cycle stages. Furthermore, in our particular study the larvae dispersed from the AE site in less than 48 hours, which indicates that while AE can attract larvae to the site, appropriate small-scale habitats must be available to retain the benefits of recruitment. Finally, challenges remain in scaling and applying AE more broadly, with two particular challenges being energy requirements and SCUBA-intensive evaluation requirements.

Our field demonstrations have shown that effective AE requires 18–20 W of continuous power during nighttime hours to broadcast reef sounds over a 42-65 m radius in Kāne’ohe Bay. Fortunately, our work has also found that AE seems required over just a few days centered on the new moon each month, which substantially reduces the long-term energy requirements per month. It may also be possible that intermittent AE broadcasts on a duty cycle may reap most the benefits of continuous playbacks while cutting power requirements further, and this is a topic for future research. Current AE setups also depend on obtaining high-quality acoustic recordings from healthy reefs within the same ecological region as deployment sites, which may limit applicability in less-studied locations. The optimal timing and tapering of acoustic broadcasts to maximize recruitment benefits while avoiding potential negative effects on resident reef organisms also requires further research.

Our work also shows promise in using autonomous cameras to evaluate the efficacy of AE without the use of extensive SCUBA operations. However, our demonstrations had three significant limitations: (1) high rates of camera failure, (2) limitations to daytime observations, and (3) inability to distinguish different species in their larval stage. Our particular choice of open-source hardware platform (the ESP32-CAM board) had poor quality control, with up to 20% of boards ordered online failing test bench quality checks. One deployment (July 2024) had over 80% of the cameras fail, even after they worked during the previous deployment. The source of the failure was traced to a particular batch of boards by a specific manufacturer. Many alternative open-source camera platforms exist, which might provide more reliable and higher-resolution camera performance^62^.

Although acoustic enrichment consistently increased larval detections relative to controls, several limitations of the current playback approach warrant consideration. Unlike natural reefs, where biological sound production is spatially distributed across broad areas of substrate, our experimental design employed a single underwater speaker acting as a point source. The camera deployments were also limited to daytime observations; the addition of external lighting would permit monitoring at night, when AE broadcasts are taking place, however this poses to risk the same invasive disruptions associated with light traps^36^. Finally, remote cameras cannot assess the species of larvae being recruited, but the combination of a camera and light traps^63^ might allow higher-resolution photo captures of individual larvae, allowing more refined taxa identification. eDNA sampling^64,65^ may also provide an additional remote sampling tool for evaluating AE performance on different species.

## Conclusion

Advancements in autonomous camera technology helped demonstrate the efficacy of acoustic enrichment in recruiting fish larvae, with treatment sites consistently outperforming control sites by 4-14 fold in terms fish larvae population proxies (MaxN), regardless of their specific location. However, AE was found to have no effect on the presence of mature fish, and fish larvae lingered for only a few days after initial visitation.

By deploying these cameras in the field, we demonstrated a cost-effective and scalable approach for collecting long-duration, high-temporal resolution data on fish recruitment patterns without the need for constant SCUBA activity. This capability is particularly valuable for assessing ecological interventions, such as AE, in remote or logistically challenging locations. The ability to monitor over multiple weeks is especially significant, as it captures biological activity aligned with key lunar cycles that influence fish larvae behaviour. By leveraging remote, long-duration monitoring, the research community can gain deeper insights into the ecological processes underpinning reef restoration, ultimately informing more effective and adaptive management practices. Further work is required to refine remote-monitoring approaches, including exploring to what extent duty-cycling can reduce long-term power requirements, and whether autonomous camera techniques can be refined to permit nighttime operation and species identification.

## Supplementary Information

Supplementary Information.

## Data Availability

All data generated in support of the findings of this study are available within the paper, its Supplementary Information and available at the following URL: https://www.oceaneboulais.net/projects/acoustic-enrichment/acoustic_enrichment_project.html
