# Supplementary material for: Autonomous cameras reveal larval reef fish responses to acoustic enrichment and lunar phase
Source: Sci Rep. 2026 Jun 26;16:21200. doi: 10.1038/s41598-026-57857-9 (PMC13346672; doi:10.1038/s41598-026-57857-9)
Supplement: Supplementary file 1 — Supplementary Information. [file 41598_2026_57857_MOESM1_ESM.pdf]

1 Supplemental Materials for *Autonomous cameras reveal larval reef fish responses to acoustic enrichment and lunar phase*

This supplement contains additional figures describing details of the artificial settlement structures (Figure S1), examples of fish larvae in photographs during all deployments at all sites (Figure S2), an example of the image processing algorithm for counting fish larvae(Figure S3), and further photographs of the experimental site layout (Figure S5) and camera equipment (Figure S6). Finally, plots of mature fish presence at the treatment and control locations are also presented, along with a statistical analysis identical to that presented in the main paper for fish larvae. (Tables 1 and 2 and Figure S8).

1.1 Statistical analysis of mature fish presence

Table 1 summarizes the camera counts (CC), MaxN, and Time Fraction metrics for mature fish for the three deployments, evaluated across the entire deployment, arranged in a format identical to Table 1 in the main manuscript. One sees that the metric values are very similar between the two sites, regardless of deployment. Table 2 plots the zero-inflated Poisson (ZIP) regression analysis for mature fish MaxN values for each deployment, in a format identical to Table 2 in the main manuscript. One sees that the 95% CI for both $b_{treatment}$ and $b_{MoonPhase}$ spans zero, indicating that no statistically significant dependence exists between MaxN value, moon phase, and AE presence. The ratio (AE multiplier) between expected values of MaxN at the treatment and control site differ little from one. No evidence exists that mature fish presence is related to moon phase, except possibly for July 2024.

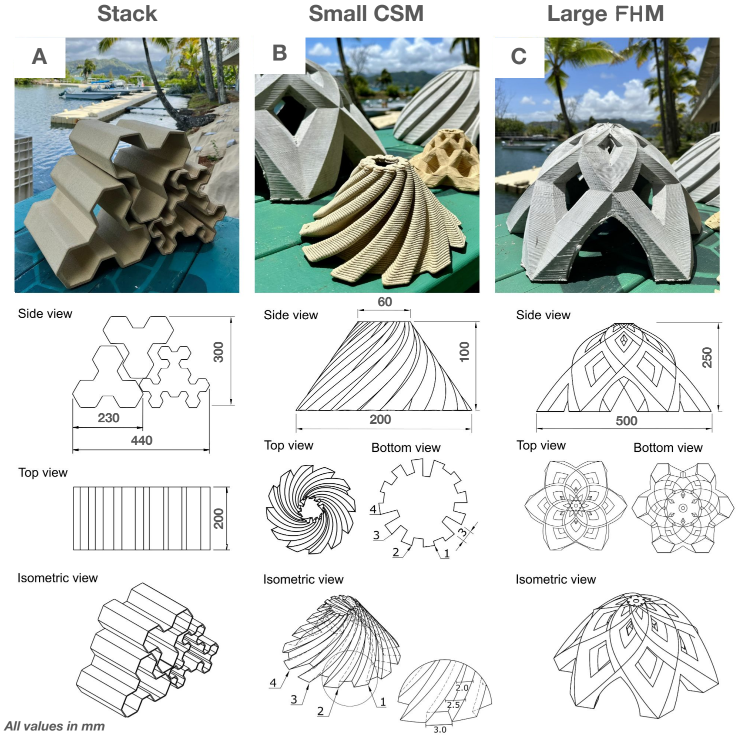

Figure S. 1. Designs of habitat structures presented in three columns: (A) stacks, (B) coral settlement modules (CSMs), and (C) fish habitat modules (FHMs). This figure shows each technical drawing with dimensions in millimeters. Stacks consist of 20 cm long hexagonal tubes of decreasing diameter (40 cm base, reducing by 8 cm each level), creating ledges and overhangs. CSM modules are conical with a 20 cm base diameter and 10 cm height, featuring four recesses of varying depths (1–4 cm) decreasing toward the top. FHMs have a dome shape (40 cm diameter, 25 cm height) with multiple 6–8 cm openings and deep alcoves (up to 8 cm) for habitat complexity. All structures were fabricated from concrete and clay to mimic natural reef complexity and provide settlement and refuge sites for reef organisms.

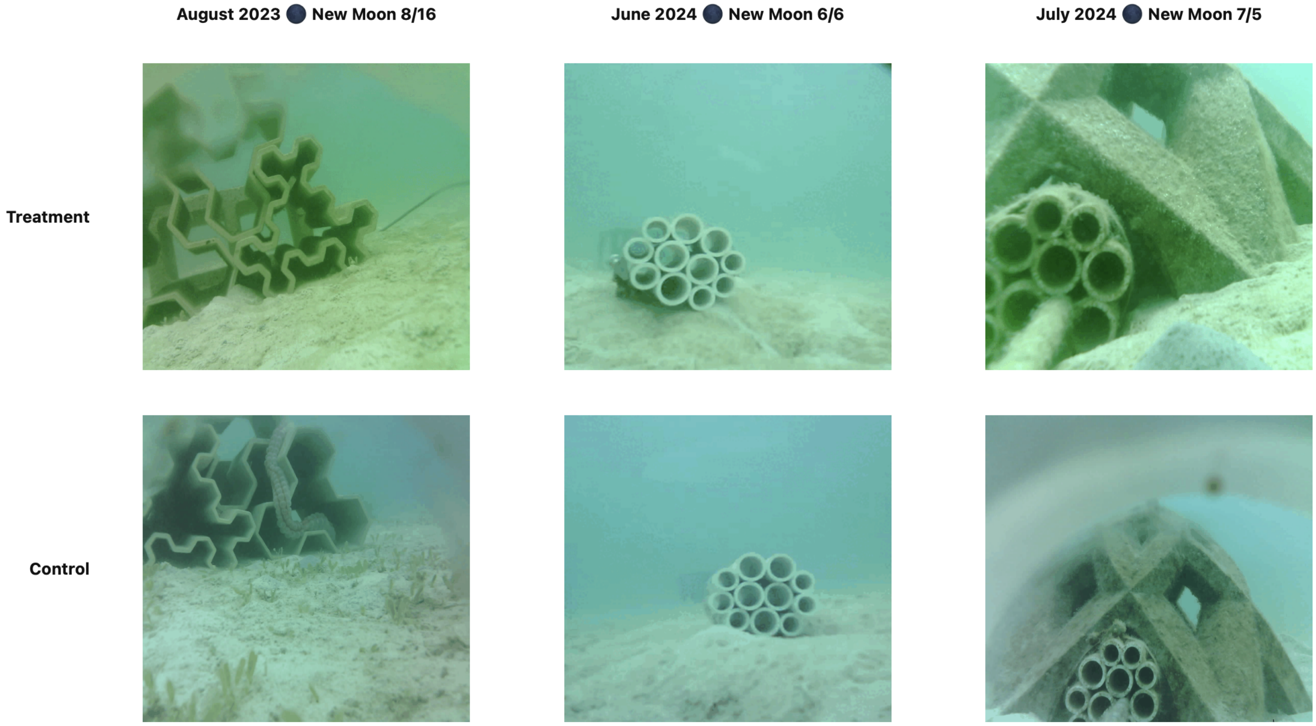

Figure S. 2. Burst-mode photographs of fish larvae presence over the three field summer deployments at treatment (top row) and control (bottom row) sites. Each image tile is associated with a moving bitmap image in Graphics Interchange Format (GIF), accessible online at this project page.

Table 1. Mature reef fish metrics computed at eastern and western sites during three deployments. “MaxN” is the largest number of fish seen in a single photograph within a 5-image burst sequence, "CC" is the average number of fish observed per camera across the entire deployment, and "TF" is the time fraction across the deployment. August 2023 numbers are a manually-counted subsample of all days. Purple highlights indicate the treatment sites.

Deployment	Cameras	MaxN (East)	MaxN (West)	CC (East)	CC (West)	TF (East)	TF (West)
August 2023	4	4	5	64.4	54.0	8.0e-3	1.4e-2
June 2024	10	4	8	30	33.6	3.8e-3	4.5e-3
July 2024	2	3	2	18	22	5.8e-4	1.6e-3

Table 2. Summary of statistical tests for mature fish during all three deployments. Results in **bold** indicate statistically significant ($p < 0.05$) differences between treatment and control sites for the rank-sum test. The AE multiplier, $e^{b_{treatment}}$ is the estimated ratio of MaxN between treatment and control sites.

**Month Year**	**Wilcoxon rank-sum test (rank sum/p-value)**			**Zero Inflated Poisson Regression (Estimate [95% CI])**		
	Counts/Camera	MaxN	Time Fraction	$b_{treament}$	$b_{MoonPhase}$	AE Mult
August 2023	275/1.0	72/0.72	74/.5725	0.17 [-0.37 0.72]	-0.48 [-2.11 1.02]	1.2
June 2024	92/0.59	83/0.91	93/0.52	-0.12 [-0.57 0.32]	-0.06 [- 0.68 0.55]	0.9
July 2024	55/0.18	61.5/0.49	53.5/0.14	-0.38 [-1.21 0.42]	**-1.88** [-3.51 -0.46]	0.7

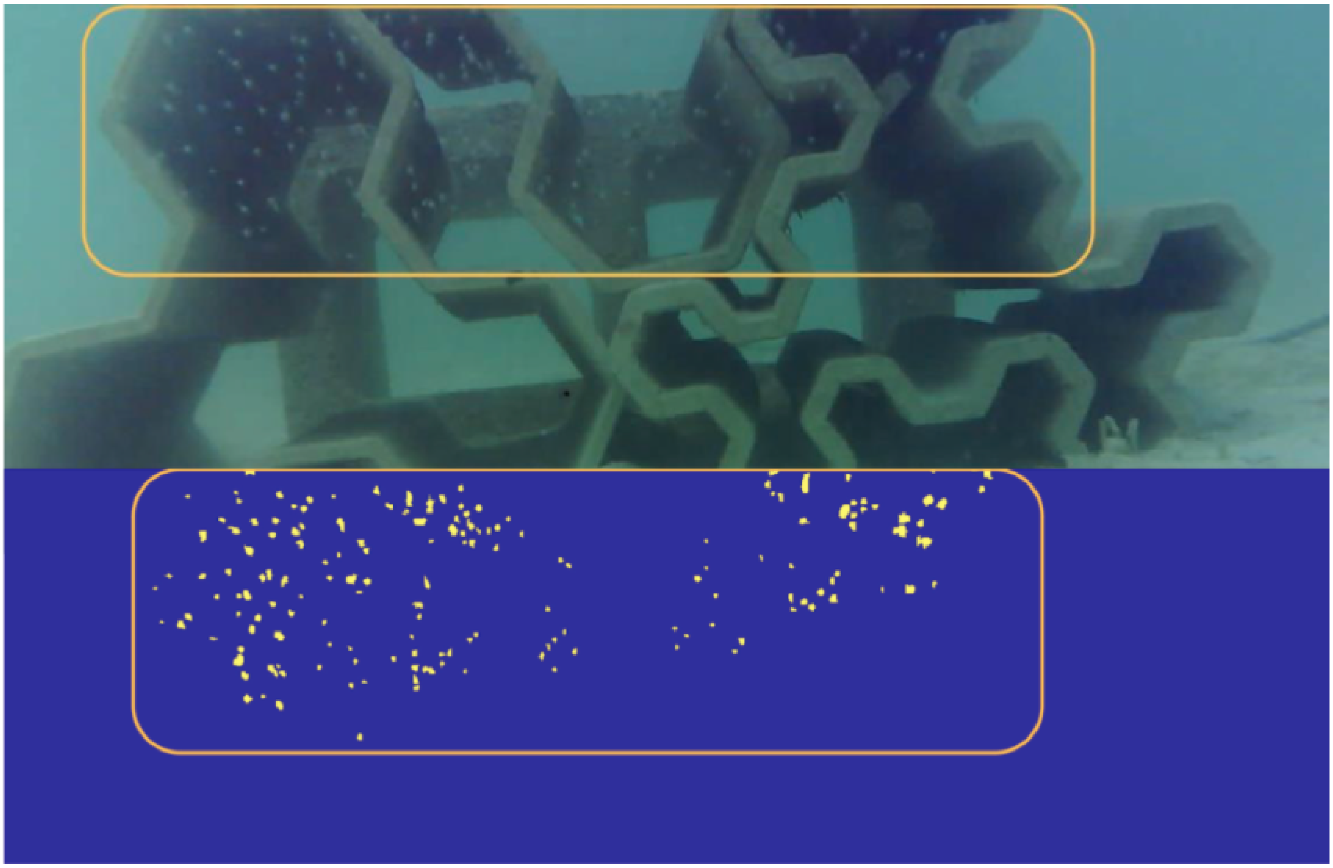

Figure S. 3. Example of binary segmentation performed on a subset of imagery from August 2023 that had > 100 larval sightings in a single frame.

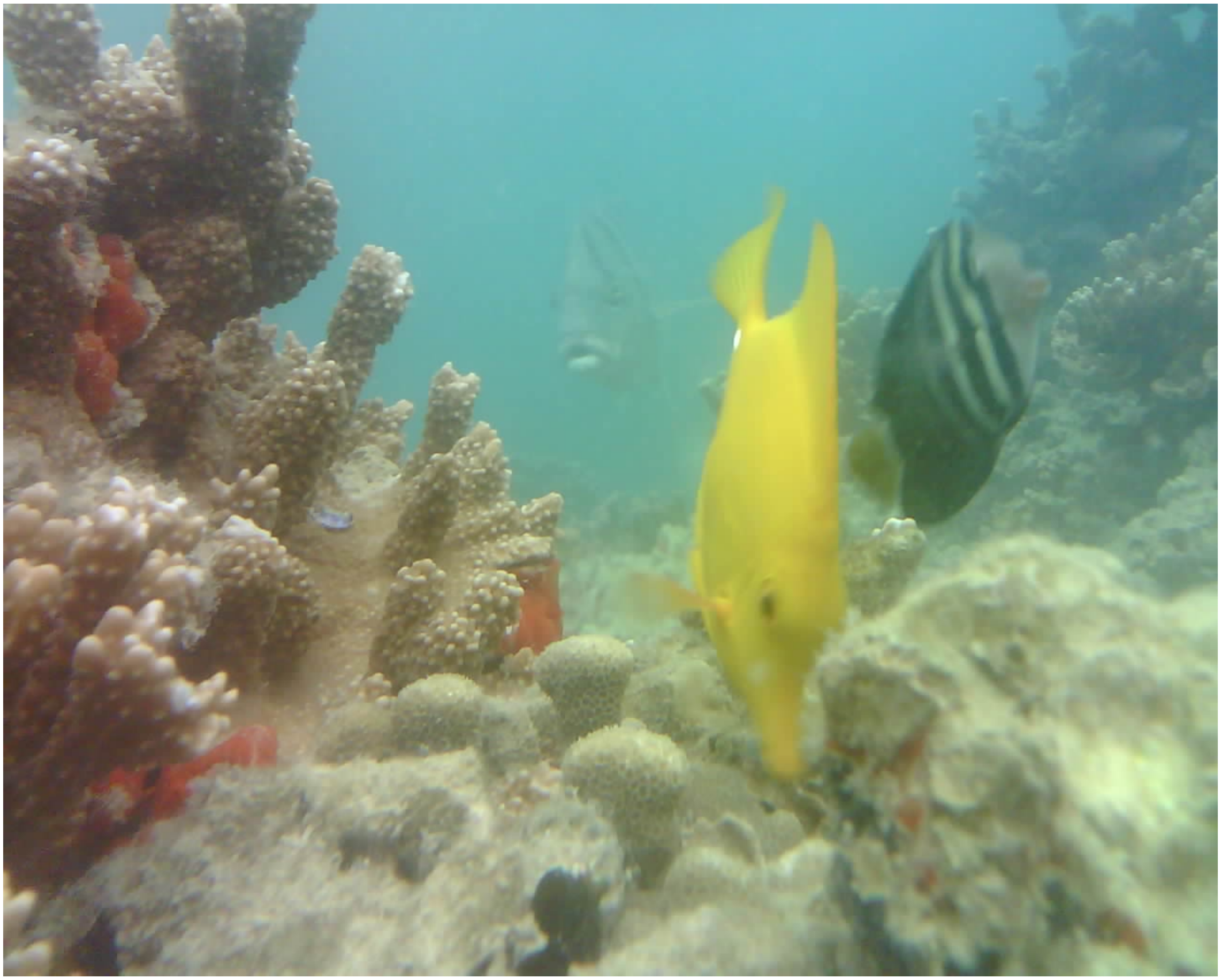

Figure S. 4. An in-situ example of the "healthy" reef site where the hydrophone was placed. This supported diverse organism assemblages, which included snapping shrimp (*Alpheus* spp.) and damselfish (*Dascyllus albisella*).

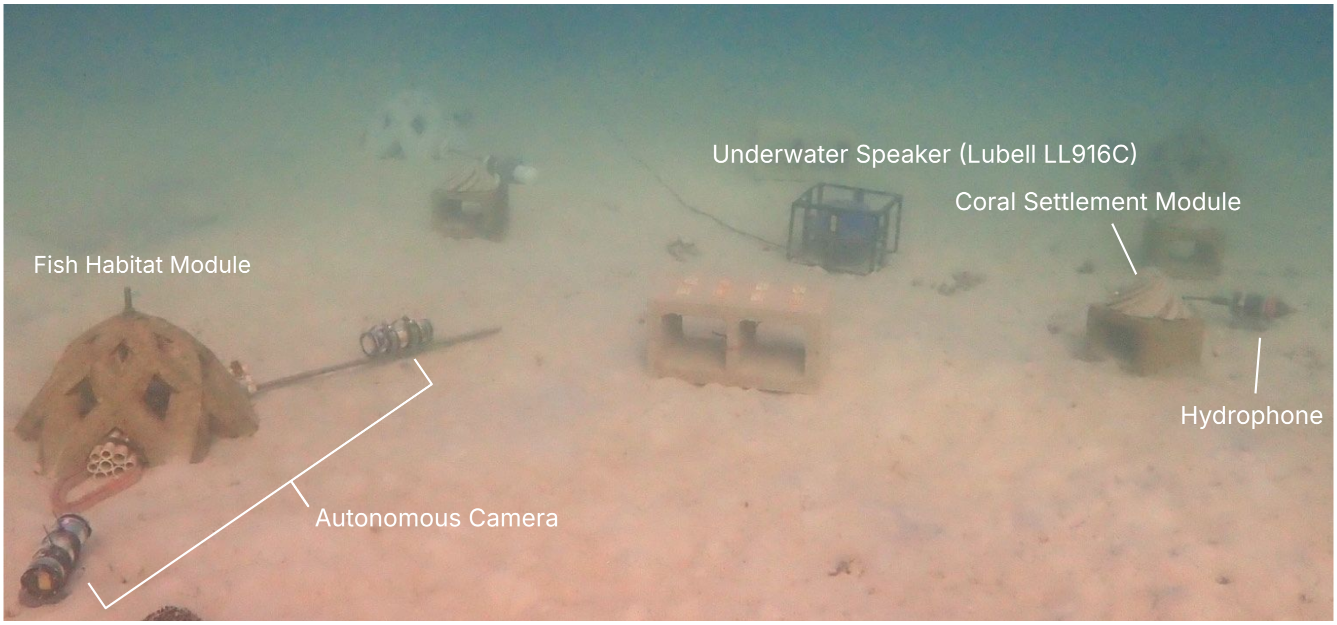

Figure S. 5. Visual layout of the equipment used at the July 2024 treatment site demonstrating (A) ceramic 3D-printed open-hexagon fish habitat structure being monitored by two (B) autonomous cameras, fitted with ESP32-S/OV2640 camera modules near the (C) activate Lubell LL916C speaker. The speaker is powered by (D) two 3.8 kWhr battery housings placed 10 m away from the site. A (E) 3D-printed superdome (CSM) structure (not used in the fish study) sits alongside a (F) single channel hydrophone that confirmed that nightly playback occurred.

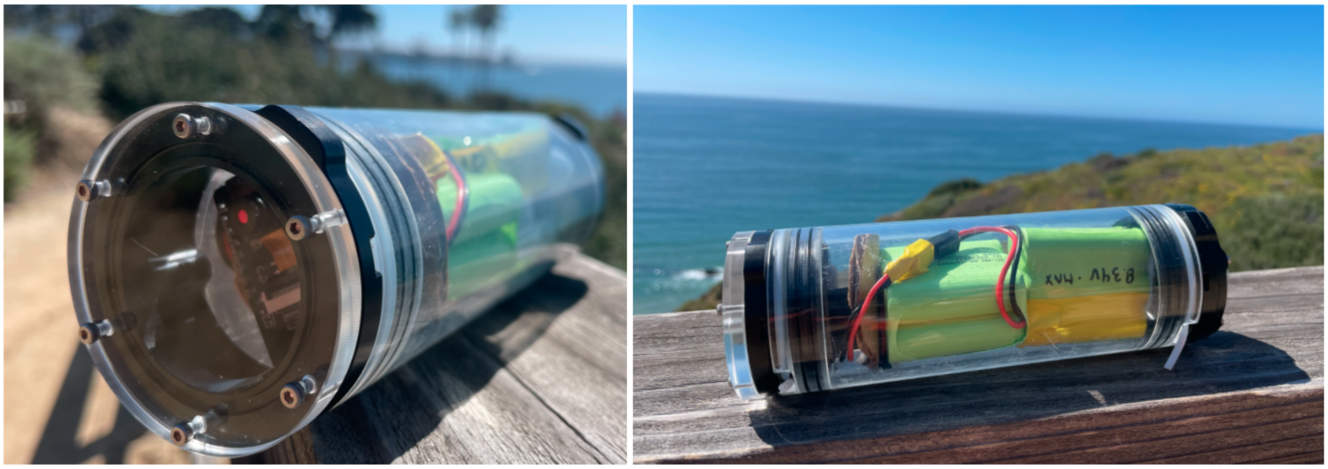

Figure S. 6. Autonomous and accessible visual monitoring: ESP32-S microcontroller with the OV2640 camera and real time clock ATMEGA325P (Ecologis Consulting, Inc) module encased in a watertight clear acrylic housing with flanged endcaps. The system is powered by two nickel metal hydride battery packs installed in a custom battery mount. All components cost 255.00 USD as of January 2025, with housing accounting for approximately 75% of the total unit cost.

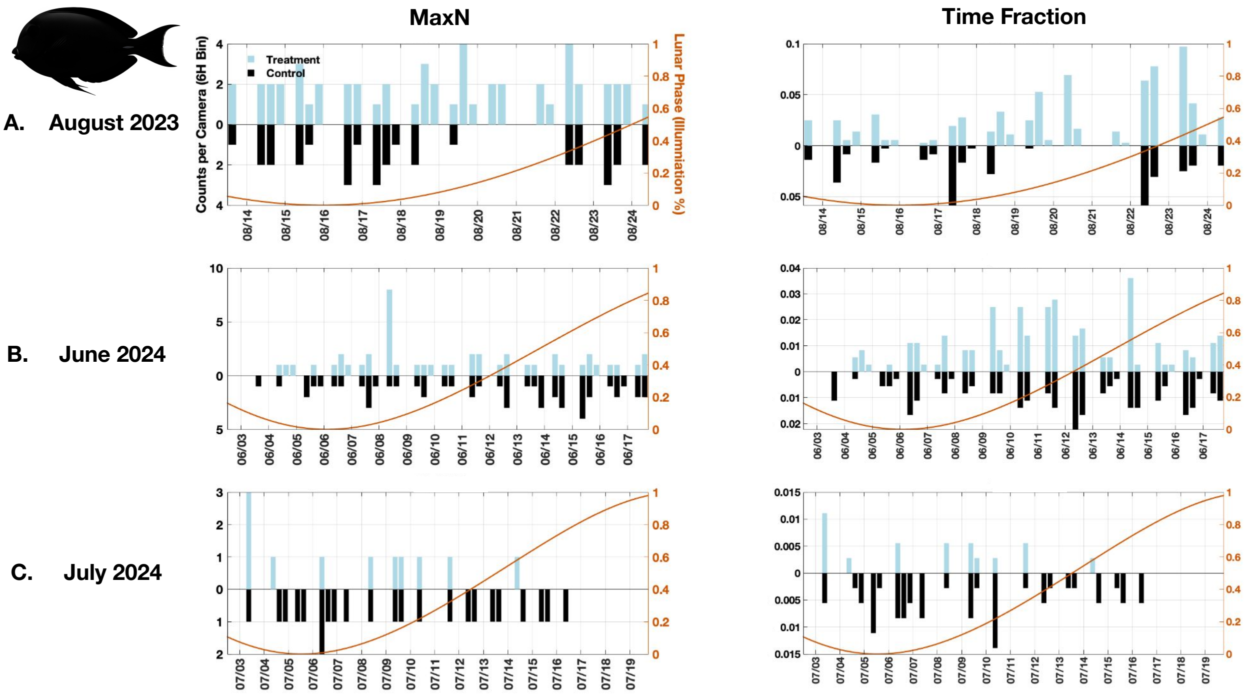

Figure S. 7. Time series for (A) August 2023, (B) June 2024, (C) July 2024 of MaxN (left column) and time fraction (right column) metrics for mature fish, evaluated every six hours during the three deployments at the treatment (blue) and control (black) sites, with control values displayed as negative values. Lunar phase (right y-axis) is displayed as an orange line.

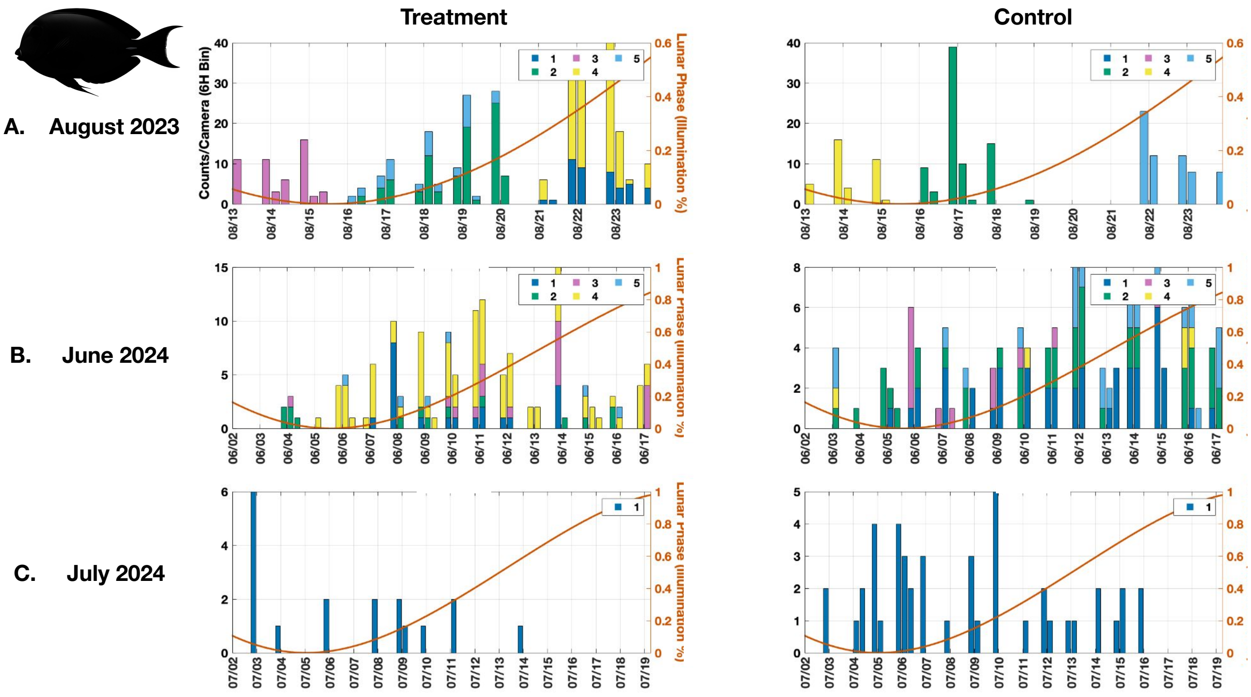

Figure S. 8. Mature fish counts per camera for August 2023 (A), June 2024 (B) and July 2024 (C) deployments, computed every six hours, with different cameras represented by unique colors. Lunar phase (right vertical scale) is displayed as an orange line. Left/right columns display results from the treatment/control site.
